# Cytokines and Myelination in the Central Nervous System

**DOI:** 10.1100/tsw.2008.140

**Published:** 2008-11-02

**Authors:** Thomas Schmitz, Li-Jin Chew

**Affiliations:** ^1^ Center for Neuroscience Research, Children's Research Institute, Washington, DC, USA; ^2^ Department of Neonatology, Campus Virchow Klinikum, Charite Universitatsmedizin, Berlin, Germany

**Keywords:** myelin, oligodendrocyte, development, demyelination, inflammation, signaling

## Abstract

Myelin abnormalities that reflect damage to developing and mature brains are often found in neurological diseases with evidence of inflammatory infiltration and microglial activation. Many cytokines are virtually undetectable in the uninflamed central nervous system (CNS), so that their rapid induction and sustained elevation in immune and glial cells contributes to dysregulation of the inflammatory response and neural cell homeostasis. This results in aberrant neural cell development, cytotoxicity, and loss of the primary myelin-producing cells of the CNS, the oligodendrocytes. This article provides an overview of cytokine and chemokine activity in the CNS with relevance to clinical conditions of neonatal and adult demyelinating disease, brain trauma, and mental disorders with observed white matter defects. Experimental models that mimic human disease have been developed in order to study pathogenic and therapeutic mechanisms, but have shown mixed success in clinical application. However, genetically altered animals, and models of CNS inflammation and demyelination, have offered great insight into the complexities of neuroimmune interactions that impact oligodendrocyte function. The intracellular signaling pathways of selected cytokines have also been highlighted to illustrate current knowledge of receptor-mediated events. By learning to interpret the actions of cytokines and by improving methods to target appropriate predictors of disease risk selectively, a more comprehensive understanding of altered immunoregulation will aid in the development of advanced treatment options for patients with inflammatory white matter disorders.

### INTRODUCTION

Cytokines are pleiotropic factors that coordinate host defenses — innate and adaptive responses — and regulate myriad other cellular functions from cell survival to maturation. The majority of cytokines are secreted proteins or glycoproteins, but some, e.g., tumor necrosis factor (TNF), are integral transmembrane proteins on the cell surface. Cytokines are produced chiefly by white blood cells, viz, lymphocytes and macrophages. Upon cellular stimulation, following mechanical injury, infection, or autoimmune activation, cytokines are synthesized at low levels and only transiently. The balance of cytokine secretion by both immune and nonimmune cells constitutes a homeostatic network that determines the outcome of cellular response. It is widely believed that, in cytokine-induced damage, immune cells (CD4/CD8 T lymphocytes, and B lymphocytes) are activated by antigen-presenting cells, resulting in the secretion of proinflammatory cytokines (e.g., interferon [IFN]-γ, interleukin [IL]-23) by T lymphocytes, which either directly affect myelin structures or activate macrophages to release nitric oxide, reactive oxygen intermediates, matrix metalloproteinases (MMPs), or TNF-α. B lymphocytes are induced to terminal differentiation by cytokines and, upon activation to release myelin-specific antibodies, induce complement-mediated demyelination. Contrary to previous belief, the central nervous system (CNS) is no longer considered an immune-privileged site with respect to the presence of immune cells and their signaling molecules. Activated glia, such as astrocytes and microglia, also secrete cytokines and express major histocompatibility complex (MHC) molecules, and blood-derived immune cells that infiltrate the CNS can further interact with astrocytes, neurons, and microglia to impact oligodendrocyte physiology and pathology.

Chemokines are small-molecular-weight chemotactic cytokines that attract leukocytes to sites of infection and inflammation[1,2]. The expression of chemokines, like cytokines, is often triggered by inflammatory mediators, such as TNF, IFN-γ, microbial toxins, or trauma, although constitutively expressed chemokines, such as CXCL1 and CXCL12, are endogenous to neurons and astrocytes, respectively[3,4]. To date, approximately 50 different human chemokines and 20 receptors have been identified, and their current nomenclature consists of a numbering system for each subfamily of ligand and receptor, based on the pattern of N-terminal cysteines in the ligand[5]. Many different chemokine ligands bind the same receptor and many ligands bind multiple receptors, and while this may permit adjustment of immune responses, very little is understood about the functional consequences of redundancy. Chemokine receptors are present not only on inflammatory cells, but also on astrocytes, oligodendrocytes, and neurons. Consequently, chemokines are involved in functions besides chemoattraction, namely neuronal development, modulation of cell adhesion, phagocytosis, T-cell differentiation, apoptosis, and angiogenesis[6,7].

### THE ROLE OF CYTOKINES AND CHEMOKINES IN CNS DEVELOPMENT AND FUNCTION

Cytokines and their receptors are expressed physiologically in CNS cells and are important for development and function of the brain, i.e., proinflammatory cytokines IL-1β, IL-2, IL-6, IL-12, IL-18, TNF-α, IFN-γ, and anti-inflammatory IL-4 and IL-10 (Table 1). Some cytokines are pleiotropic, such as IFN-β, and even proinflammatory cytokines possess anti-inflammatory properties and vice versa. Both infiltrating immune cells and resident glial cells whose activities are associated with inflammation may also contribute to repair and regeneration through the secretion of neuroprotective factors, such as leukemia inhibitory factor (LIF), transforming growth factor-β (TGF-β) (Table 1), and ciliary neurotrophic factor (CNTF).

Astroglia, microglia, oligodendrocyte progenitor cells (OPCs), and differentiated oligodendrocytes express **IL-1β** and IL-1β receptor protein constitutively in the CNS[8]. IL-1β can inhibit proliferation of late progenitor oligodendrocyte (O4+), but has no effect on proliferation in early progenitor cells (A2B5+). IL-1β is expressed at high levels in the CNS during pre- and postnatal development[9,10], but only at low levels in adult CNS[11]. A crucial effect on glial cells in the process of myelination has been suggested by experiments in adult IL-1β knockout mice in which remyelination after brain injury did not occur[12].

**TNF-α** is mostly known to exert inflammatory and neurotoxic effects, but is also involved in normal development and function of the brain. TNF-α is highly expressed in the embryonic brain[13]. However, mutant mice that do not express TNF-α-R1 and TNF-α-R2 (TNFR1, TNFR2)develop morphologically normal brains and display normal behavior[14], as well as do TNF-α knockout mice[15]. Interestingly, in a more recent study, mice that lacked either TNF-α or TNFR2 had impaired remyelination in a toxin-induced

**TABLE 1**
**Cytokines and Chemokines Associated with White Matter Damage**

| Cytokine/ Chemokine | Effect* | Ref. |
|---|---|---|
| IL-1β | IL-1β is a growth factor for *in vivo* astrocytes during brain development. | [223] |
| | IL-1β and IL-1β receptor are constitutively expressed in the CNS by astroglia, microglia, OPCs, and differentiated oligodendrocytes. IL-1β inhibits OPC proliferation and enhances survival and differentiation to MBP+ stage. | [8] |
| | In IL-1ra knockout mice, astroglial activation after TBI was attenuated. | [191] |
| | In adult IL-1β knockout mice after brain injury, remyelination did not occur. | [12] |
| | Brain injury caused by high oxygen in newborn rats is accompanied by increased expression of IL-1. | [186] |
| | Levels of IL-1β in the CSF of patients after TBI are related to clinical outcome. | [123,127] |
| | Genetic variants of IL-1β are associated with clinical outcome in head trauma patients. | [130] |
| | In patients after TBI, increasing IL-1ra is associated with decreasing intracranial pressure. | [129] |
| | Polymorphisms of IL-β or IL-1R have been reported as being associated with increased risk of stroke in some studies. | [58,59] |
| | In a large cohort study (Framingham Study), high plasma levels of IL-1β were related to development of AD. | [99] |
| | Childhood and adult schizophrenia are accompanied by elevated IL-1. | [108,109] |
| | IL-1 is elevated after exposure to hyperoxia *in vivo* in neonatal rodent and sheep models. | [186,188] |
| IL-2 | IL-2 is highly expressed in white matter lesions in the brains of premature infants. | [231] |
| IL-6 | High serum levels of IL-6 are associated with development of WMD in preterm infants. | [54] |
| | IL-6 up-regulation correlates with better outcome in children with severe TBI. | [232] |
| | In newborn rats, IL-6 neutralizing antibodies are associated with attenuated brain injury caused by LPS. | [233] |
| | In newborn mice, IL-6 increased astrogliosis after TBI. | [234] |
| | IL-6 promotes tissue repair following TBI in mice. | [235] |
| | IL-6 promotes neuron and oligodendrocyte survival in the presence of glutamate excitotoxicity. | [236] |
| | IL-6 receptor/IL-6 fusion enhances differentiation of OPCs and promotes oligodendrocyte survival. | [237] |
| IL-10 | IL-10 attenuates white matter injury in neonatal rats caused by maternal infection. | [238] |
| | Treatment with IL-10 after onset of inflammation is neuroprotective in the newborn piglet brain. | [31] |
| IL-12 | In a nonhuman primate model of MS, disease suppression is observed after anti–IL-12 antibody administration. | [239] |
| | No association of peripheral IL-12 levels after birth and development of WMD in preterm infants. | [54] |
| | In newborn sheep, IL-12 was increased in the subcortex after resuscitation with 100% oxygen compared with 21% oxygen. | [188] |
| IL-18 | Administration of IL-18 binding protein leads to decreased intracranial IL-18 levels and to improved neurological outcome in mice after experimental closed head injury. | [190] |
| | IL-18 is increased after hypoxia/ischemia; mice deficient for IL-18 had attenuated brain lesions. | [170] |
| | IL-18 is elevated after exposure to hyperoxia *in vivo* in neonatal rodent and sheep models. | [186,188] |

*Table 1 continues*

**TABLE 1 (continued)**

| | | |
|---|---|---|
| | Elevated IL-18 is associated with cystic white matter lesions in preterm infants with posthemorrhagic hydrocephalus. | [55] |
| IFN-β | IFN-β knockout mice show accelerated remyelination in the cuprizone animal model of demyelination. | [162] |
| | IFN-β attenuates apoptosis of fetal and neonatal rat astrocytes *in vitro.* | [240] |
| | No cytoprotective effect of IFN-β on oligodendroglia injury induced by $H_2O_2$, NO, complement, or glutamate. | [216] |
| | No effect of IFN-β *in vitro* on survival, proliferation, and migration of rat OPC cell line. | [30] |
| | IFN-β antagonizes IFN-γ–induced up-regulation of class II expression. | [29] |
| | IFN-β causes down-regulation of MHC class II antigens and of leukocyte influx into the CNS. | [241] |
| IFN-γ | IFN-γ protects against demyelination in a cuprizone animal model. | [23] |
| | In cuprizone-induced demyelination, IFN-γ inhibits remyelination through endoplasmic reticulum stress. | [242] |
| | Overexpression of IFN-γ in transgenic mice leads to demyelination. | [21] |
| | High levels of IFN-γ in the serum and in the CSF of preterm infants correlate to WMD. | [54,55] |
| | IFN-γ inhibits OPC differentiation and oligodendrocyte progenitor cell cycle exit. | [24,26] |
| | IFN-γ expression is increased in WMD in the human brain. | [243] |
| | IFN-γ is elevated *in vivo* in neonatal sheep after exposure to hyperoxia. | [188] |
| TNF-α | TNF-α highly expressed in embryonic brain. | [13] |
| | TNF-α knockout mice have normal neurological development. | [15] |
| | After ischemic injury, TNF-α knockout mice have increased neuronal damage. | [14] |
| | *In vitro*, TNF-α induces proliferation of astrocytes. | [18] |
| | *In vitro*, TNF-α induces synthesis of NGF. | [19] |
| | TNF-α is up-regulated in MS lesions and expressed by macrophages and astrocytes in active lesions in humans. | [244] |
| | TNF may attenuate inflammation in MS; in TNF knockout mice, immunization with myelin oligodendrocyte glycoprotein leads to high mortality, extensive brain inflammation, and demyelination. | [245] |
| | In murine experimental autoimmune encephalitis, TNF blockade reduced MS lesions. | [156] |
| | Treatment with TNF antibody increased inflammation in patients with MS. | [157] |
| | TNF-α causes apoptosis of oligodendrocytes. | [246,247] |
| TGF-β | TGF-β cytokines are highly expressed by microglia and astroglia in demyelinating areas in MS. | [248] |
| | Decreased TGF-β in human poststroke brain tissue is associated with microangiopathy in the white matter. | [242] |
| | TGF-β reduces demyelination in viral model of MS via immunomodulatory mechanisms. | [249] |
| | Cytotoxicity of activated microglia against oligodendrocytes *in vitro* is attenuated by TGF-β. | [250] |
| | TGF-β inhibits proliferation of OPCs and promotes oligodendrocyte development. | [251] |
| LIF | LIF receptor signaling enhances oligodendrocyte survival and limits demyelination in murine autoimmune encephalomyelitis. | [33] |
| | Exogenously administered LIF limits cuprizone-induced demyelination; LIF knockout mice exhibit potentiated demyelination and oligodendrocyte loss after cuprizone challenge. | [252] |
| | LIF prevents oligodendrocyte apoptosis through induction of JAK/STAT and Akt pathways. | [253] |

*Table 1 continues*

**TABLE 1 (continued)**

| | | |
|---|---|---|
| | Astrocytes *in vitro* promote myelination in response to electrical impulses via expression of LIF. | [32] |
| | LIF promotes oligodendrocyte maturation, but directs differentiation of OPCs into astrocytes in the presence of extracellular matrix. | [254,255] |
| CXCL1 | Endogenously produced in neurons and astrocytes. | [3] |
| | Inhibits migration of oligodendroglia precursor cells into the white matter tract. | [45] |
| | Stimulates MBP production in cultured oligodendrocyte cell lines. | [46] |
| CCR1 | Due to EAE animal models, CCR contributes to disease severity in MS. | [151] |
| CCR2 | CCR2 expression is elevated in T cells from patients with SP MS than in RR MS. | [86] |
| | Contribution to severity of MS in animal models. | [152] |
| | In mouse models, altered CCR2 is associated with early disease progression of AD. | |
| CCL2 | Serum and CSF levels of CCL2 are lower in patients with SP MS than in RR MS. | [82] |
| | In animal models, expression of CCL2 RNA increases significantly in the cortex after stab wound and cryolesion. | [256] |
| | CCL2 was found in mature, but not in immature, senile plaques. | [104] |
| CCR5 | Expression on macrophages and microglia are associated with early remyelination in patients with MS. | [85] |
| | Immunomodulatory MS therapies cause modulation of CCR5 expression in T cells. | [92] |
| | CCR5 32-bp deletion was found associated with late-onset schizophrenia. | [116] |
| CCL11 | Increase of CCL11 in patients with schizophrenia compared to controls. | [115] |
| CXCL12 | CXCL12 knockout mice develop brain abnormalities. | [38] |
| | Endogenously produced in astrocytes; stimulates astrocyte proliferation. | [4] |

* Definitions for abbreviations may be found in the text.

model of demyelination[16]. After ischemic injury, TNF-α knockout mice have increased neuronal damage[14], indicating *in vivo* neuroprotection mediated via TNF-α-R. Astrocytes and oligodendrocytes express predominantly TNF-RI, meanwhile microglia express both TNF-α-RI and TNF-α-RII[17]. In primary astrocytes, TNF-α induces proliferation[18] and the synthesis of neurotrophic factors, such as neural growth factor (NGF)[19]. In CNS neurons, TNF-α seems to be involved in neuritic process outgrowth[20].

**IFN-γ** is a major cytokine involved in demyelinating pathologies and there are little data on its potential role in physiologic development and function of the CNS. In IFN-γ transgenic mice, retinal development is grossly altered, suggesting a role in nerve development[21]. While targeted overexpression of IFN-γ in white matter of transgenic mice results in myelin ablation[22], evidence from a toxic demyelination model suggests that low levels of IFN-γ may also have surprisingly important roles in protection against severe myelin loss[23]. *In vitro*, purified oligodendrocytes treated with IFN-γ undergo apoptosis or necrosis[24], and OPCs display altered cell metabolism[25] and cell cycle characteristics[26]. Cell surface expression of MHC class I molecules are strongly stimulated by IFN[27]. Some effects of IFN-γ on OPCs, including reduced myelin gene expression[25,26] (Fig. 1), may be associated with the IFN-induced accumulation of MHC I molecules[28].

Although the beneficial effect of **IFN-β** in preventing deterioration of demyelinating diseases is well documented, little is known about its mechanism of action on myelination in the CNS. So far, mainly immunomodulatory processes have been identified, including down-regulation of MHC class II antigens and of leukocyte influx into the CNS. Regarding CNS-intrinsic actions, IFN-β has been demonstrated to promote survival of astrocytes *in vitro*. Moreover, IFN-β antagonizes IFN-γ–induced up-regulation of class II expression[29]. Data on interactions between IFN-β and oligodendrocytes are hardly available; so

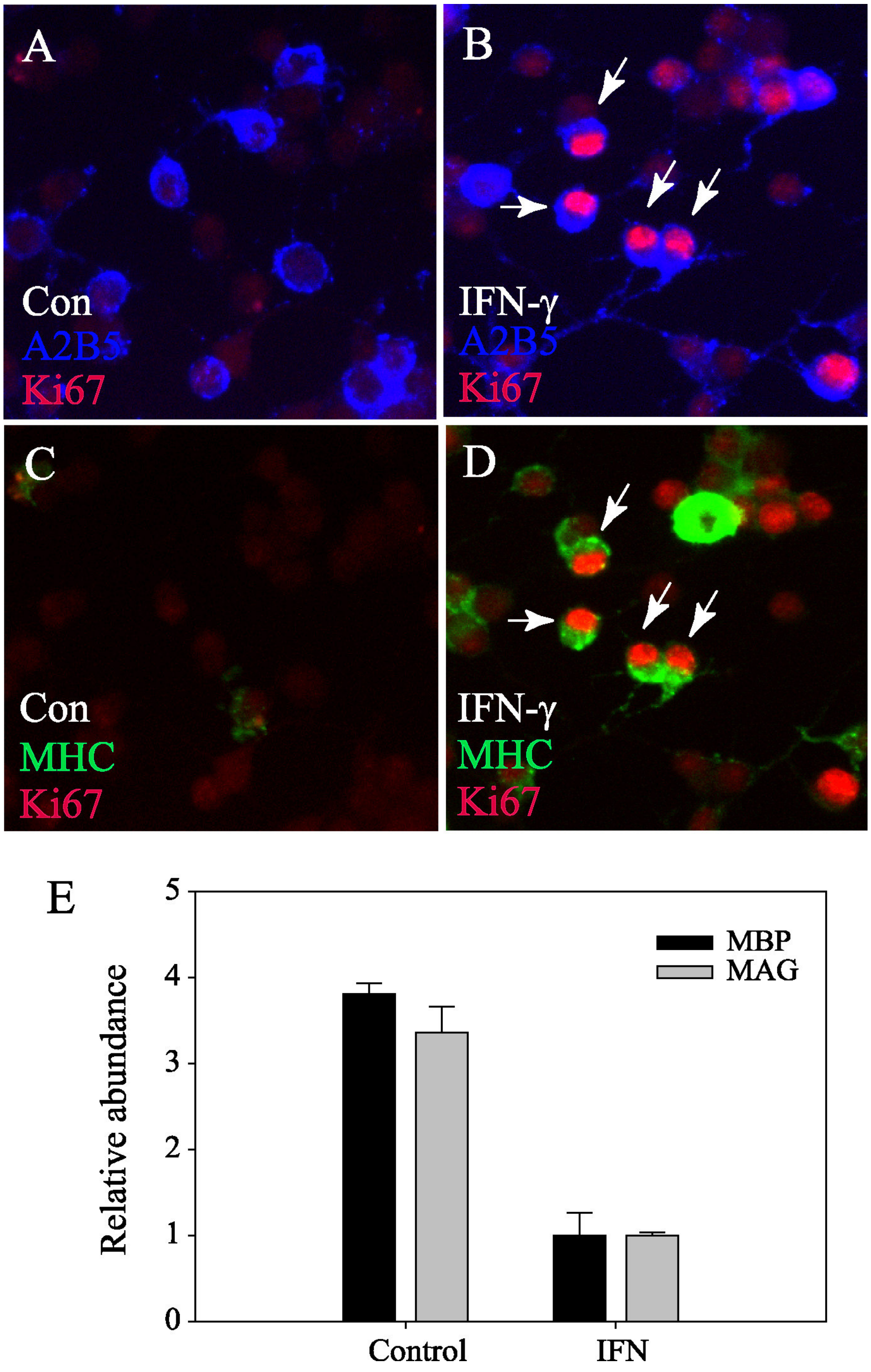

**FIGURE 1.** Effects of IFN-γ on proliferation, MHC, and myelin gene expression in differentiating OPCs in culture. (A–D) IFN-induced MHC expression is colocalized with cell cycle marker Ki67 in A2B5+ OPCs. Triple immunofluorescent labeling of OPCs maintained in culture with 30 ng/ml thyroid hormone (T3) for 4 days in the absence (A,C) and presence (B,D) of 15 ng/ml rat IFN-γ. Dual-labeled cells are shown in pairs, with A,B showing Ki67 (red) and A2B5 (blue) labeling, while C,D show Ki67 and MHC I (green). Ox18 labels conserved parts of the heavy chain regardless of MHC haplotype. White arrows indicate triply labeled A2B5+, MHC+, and Ki67+ cells. Magnification ×400. (E) Reverse transcription-quantitative (RT-qPCR) PCR analysis of total RNA for myelin basic protein (MBP) and myelin-associated glycoprotein (MAG) from OPCs cultured in the absence (control) and presence (IFN) of 15 ng/ml IFN-γ for 3 days in T3.

far, no effect on survival, proliferation, and migration of a rat OPC cell line was observed in *in vitro* experiments[30].

The anti-inflammatory cytokines **IL-4** and **IL-10** have been found repeatedly to be increased in distinct entities of CNS inflammation. IL-10 can be produced by macrophages, B cells, and Th2 cells in addition to astrocytes and microglia. As an immunomodulator, IL-10 inhibits Th1 proliferation and effector cytokine production. A neuroprotective effect of IL-10 has been documented in rats receiving IL-10 either intracranially or into the peritoneum during brain injury[31].

**Leukemia inhibitory factor (LIF)** is protective in trauma, but in response to neuronal input, was also recently shown to promote myelination in cortical/glial cocultures. The expression of LIF mRNA was up-regulated in astroglia in response to neuronal activity[32]. A concomitant increase in myelination after electrical activation occurred in cocultures from wild-type mice, but not in those from LIF knockout mice. Furthermore, LIF-deficient mice exhibit fewer glial fibrillary acidic protein (GFAP)–positive astrocytes and lower levels of MBP. Several molecules, including **CNTF**, that act through the LIF receptor or gp130 signaling chain have neuroprotective actions on either neurons or oligodendrocytes, and lead to enhancing oligodendrocyte survival in the face of inflammatory cytotoxicity[33].

**Chemokines and their receptors** are associated with a great number of pathologies, including autoimmune disorders and neurodegenerative diseases, such as Alzheimer's disease (AD), meningitis, HIV encephalitis, brain trauma, contusion injury, and multiple sclerosis (MS)[7,34]. HIV gp120–induced apoptosis in neurons is prevented by CX3CL1, CXCL12, CCL3, and CCL5, and glutamate-induced damage is prevented by CXCL8, CXCL12, CCL5, and CCL2[35,36,37].

**CXCL12,** via CXCR4 activation, is crucial for CNS development, as brain abnormalities are apparent in knockout mutants[38]. CXCL12/CXCR4 regulates the migration of cortical neuronal precursor cells, and in its absence, an ectopic dispersion of Cajal-Retzius cells are displaced deeper into the cortical layers[39]. Both CXCL1 and CXCL12 are expressed by astrocytes, and the latter stimulates astrocyte proliferation, chemotaxis, and cytokine and chemokine production[40,41,42].

There is little evidence that oligodendrocytes produce chemokines, but it has been shown that astrocytic **CXCL1** in the spinal cord enhances the proliferative response of OPCs to their mitogen platelet-derived growth factor (PDGF)[43]. This would account for the association of increased numbers of OPCs with elevated levels of CXCL1 in the dysmyelinating mutant jimpy mouse[44]. CXCL1, signaling through CXCR2, inhibits the directed migration of OPCs into the white matter, indicating that CXCL1 is required for the population of the presumptive white matter by oligodendrocyte precursors[45]. In addition, CXCL1 and CXCL12 both stimulated the proliferation of an OPC-like line, and promoted MBP synthesis in primary myelinating cultures[46]. The importance of signaling through CXCR2 was underscored in CXCR2-ablated mice, in which spinal cord white matter area, myelin sheath thickness, levels of myelin proteins MBP, proteolipid protein (PLP), and astrocytic GFAP were reduced, along with a decreased rate of conduction by spinal cord axons[47]. Taken together, these results indicate that CXC chemokines play critical roles in the normal development of white matter tissue and, hence, in the maintenance of axonal function.

### INFLAMMATION IN THE CONTEXT OF WHITE MATTER DISEASE AND IN MENTAL DISORDERS

#### Inflammation Contributes to White Matter Damage in Newborn Infants

White matter damage (WMD) in infancy and early childhood is observed in the context of pronounced prematurity, in association with complex congenital heart diseases and in genetic and metabolic disorders (i.e., leukodystrophies). In preterm infants, pathogenesis of WMD is still incompletely understood. Clinically, it is associated either with diffuse vanishing of the white matter plus dilation of the ventricles or with focal/cystic lesions, and it is recognized as a strong predictor of cerebral palsy[48]. Hypoxia/ischemia[48], chronic hypoxia[49], and infection and inflammation involving proinflammatory cytokines[50] have been described to be associated with WMD in infants. In clinical studies, levels of proinflammatory cytokines (i.e., IL-1β, IL-6, IL-18, IFN-γ) in amniotic fluid, umbilical cord blood, and cerebrospinal fluid (CSF), as well as the presence of chorioamnionitis and fetal vasculitis, correlate with the development or presence of WMD and impaired neurologic development in preterm infants[51,52,53,54,55].

### The Role of Cytokines in Stroke and Hypoxia/Ischemia

Cerebral ischemia initiates an inflammatory response in the brain that is associated with induction of a variety of cytokines, including TNF-α, IL-1β, and IL-6. Several studies have focused on the role of IL-6 as an early signal of the inflammatory response after acute ischemic stroke. Levels of IL-6 in the serum and in the CSF of acute stroke patients have been reported to correlate with brain infarct volume, stroke severity, and clinical outcome[56,57]. In some studies, genetic variants of IL-1β and IL-1R have been associated with higher risk of stroke[58], although these data were not confirmed in a large prospective study just published[59]. LIF, too, may play a role in postischemic inflammation since increased LIF expression was detected in peri-infarcted regions in an autopsy specimen of acute stroke patients[60] and in a rat model of middle cerebral artery occlusion[61].

Asphyxia and hypoxic/ischemic insults are predisposing factors for WMD in neonates[62]. An intrinsic vulnerability of OPCs is considered central to the pathogenesis of WMD, and the release of proinflammatory cytokines TNF-α and IL-6 may contribute to the susceptibility of these cells. In neonates after asphyxia, inflammatory reactions occur, including elevated cytokine production. Serum concentrations of IL-6, IL-8, and IL-10 were higher in asphyxiated neonates than in the normal neonates[63]. High levels of IL-1β, IL-6, and TNF-α in the CSF of asphyxiated neonates correlated with clinical scores of hypoxic/ischemic encephalopathy. Abnormal neurological development of these neonates at 6 and 12 months of age were predicted by high IL-1β in the CSF.

### Inflammation is an Important Pathogenetic Factor in Multiple Sclerosis

Multiple sclerosis (MS) is a disease of young adults, characterized by clinical exacerbation, inflammation, demyelinated plaques in the brain and spinal cord visible by magnetic resonance imaging (MRI), and neurological deficits. It is believed to be an immune-mediated disorder occurring in genetically susceptible individuals, and CSF from patients with aggressive MS has been shown to cause axonal damage and neuronal apoptosis *in vitro*. However, the events leading to the initiation of disease remain largely unknown. Several patterns of MS have been distinguished: relapsing remitting (RR), primary progressive (PP), secondary progressive (SP), and progressive relapsing (PR) MS[64]. Given the heterogeneous nature of disease presentation, the measurement of cytokine levels has often produced confusing results and has had limited use in diagnosis. Nonetheless, despite difficulties in extrapolating experimental findings from EAE (see below) to produce clinical improvement, the modulation of cytokine function in MS using glatiramer acetate (Copaxone) has been shown to be effective, indicating that cytokine-mediated activities remain important therapeutic targets. TNF-α in the CNS is significantly up-regulated in MS lesions and is expressed by macrophages, microglia, and astrocytes in chronic active lesions. Several investigators have found positive correlation between TNF-α and clinical course. Levels of TNFR p55 are also higher in patients with stable MS. In MS patients, IFN-γ and IL-10 secretion are increased, and progressive disease was more frequent in carriers of the IFN-γ R2 allele Arg64[65]. In addition to TNF and IFN, IL-6 and IL-18 are also up-regulated in CNS and CSF, respectively, along with the detection of decreased IL-10 production by peripheral blood mononuclear cells (PBMCs) before the onset of exacerbation.

Current MS treatments may be classified into agents for acute relapses, such as corticosteroids, e.g., methylprednisolone, or disease-modifying agents (DMAs). IFN-β1a (Serono, Inc., Rockland, MA), IFN-β1b (Berlex Laboratories, Montville, NJ), and glatiramer acetate are currently available DMAs. As a first-line treatment for RR MS, subcutaneous IFN-β injection has been shown to reduce the frequency and severity of clinical relapses, slowing the progression of disability and suppressing signs of disease activity assessed by MRI detection[66,67]. IFN-β therapy, but not glatiramer acetate, was able to increase the time to disease progression[68,69]. However, many patients continue to experience disability progression despite the use of these more established DMAs.

Integrins are a family of surface adhesion molecules that aid in the adhesion and migration of immune cells. The glycoprotein $\alpha_4$-integrin is expressed at high levels on the surface of leukocytes, which contributes to firm adhesion to the endothelium[70]. Natalizumab is a humanized monoclonal antibody against the $\alpha_4$ subunit of integrins and blocks binding to their endothelial receptors, vascular cell adhesion molecule-1 (VCAM-1), and mucosal addressing-cell adhesion molecule 1 (MadCAM-1). This impairs the migration of lymphocytes through the blood-brain barrier (BBB) into the brain parenchyma, suppressing inflammation in EAE[71,72]. Natalizumab may also inhibit ongoing inflammatory responses by interrupting the interaction between integrin-expressing T lymphocytes and extracellular matrix proteins, such as fibronectin and osteopontin. In several multicenter trials[73,74,75], natalizumab significantly reduced the rate of clinical relapses, the risk of progression of disability, and the number of new or enlarging brain lesions on MRI, showing improvement even with visual outcome[76]. Although natalizumab was shown to reduce relapse rates and lesions in patients taking IFN-β, two cases of progressive multifocal leukoencephalopathy (PML) have also occurred in these patients. A recent study has revealed that natalizumab treatment does not inhibit systemic cytokine expression, as treatment had instead raised the levels of TNF-α mRNA and number of cells secreting TNF-α and IFN-γ over 6 months[77]. This correlates with the lack of effect of natalizumab on MS fatigue, which is associated with increased energy consumption due to neuronal damage, cytokine effects, and depression[78]. For treatments involving IFN-β and natalizumab, the development of neutralizing antibodies can interfere with the biological response of these agents. This is not an issue for immunomodulatory agents like glatiramer acetate, which still maintains a place among first-line treatments.

In addition to targeting T cells with IFN-β and glatiramer acetate, there is increasing evidence that B cells also contribute to MS pathogenesis. Rituximab, a genetically engineered chimeric monoclonal antibody, depletes CD20+ B lymphocytes through a combination of cell-mediated and complement-dependent cytotoxicity. Phase I and II trials with RR MS showed promising outcomes in reducing relapses[79,80,81], although opportunistic infections have been reported. Nonetheless, anti-CD20 agents remain an option provided long-term trials for safety and efficacy prove favorable.

Analysis of MS tissue has revealed elevated levels of CXCL9, CXCL10, CCL2, CCL7, and CCL8 in reactive astrocytes within demyelinating lesions[82,83]. Some receptors (such as CXCR3 on lymphocytes) correlate with tissue destruction[84], while others (such as CCR5 on macrophages and microglia) are associated with early remyelination[85]. CCR2 expression is elevated in T cells from patients with SP MS more so than in those with RR MS[86], and several studies have shown that both serum and CSF levels of CCL2 are lower in RR MS patients[82]. CCR2+ cells migrate across a BBB model with greater efficiency than CCR2– cells [87], suggesting that CCL2/CCR2 may be important in the pathogenesis of MS.

Oligodendrocyte lineage cells express a number of CXCRs: CXCR1, CXCR2, and CXCR3, but their ligands CXCL1, CXCL8, and CXCL10 were only detected in hypertrophic astrocytes around active MS lesions[88]. Cultured human astrocytes could be induced by the proinflammatory cytokines IL-1β and IFN-γ to express these ligands at high levels, and although CXCR expression was constitutive on oligodendrocyte cells, higher levels were also observed in MS tissue. CXCL1 has been shown to enhance the effects of PDGF on NG2+ oligodendrocyte progenitors[43]. Although the effects of CXCL8 and CXCL10 in oligodendrocyte cells have not been fully characterized, CXCL8 and CXCL10 have been shown in other cell types to induce proliferation of endothelial cells and melanocytes[89,90]. This suggests that the expression and induction of chemokines and their receptors could underlie some of the observations of increased OPC numbers around active MS lesions[44].

Immunomodulatory MS therapies employing methylprednisolone have been shown to modulate chemokine production[91] and CCR5 expression in T cells[92], and IFN-β reduces the production of CXCL8, CXCL9, CXCL10, CCL2, CCL5, and CCL7 in PBMCs[93,94]. Chemokine ligands have been investigated as pharmaceutical targets, and small molecule inhibitors are being developed for a wide range of disease applications, particularly as inhibitors of HIV entry, and for other autoimmune diseases. Of the CCR1 antagonists reported, BX471 is the most potent, showing activity in rat EAE models[95]. However, BX471 failed to block immune-cell infiltration and formation of new active lesions substantially in an MS trial. As with CCR2 antagonists like INCB3344, the unexpected challenge appears to be species specificity of these molecular agents, which potentially limits the applicability of this approach for neurodegenerative disease.

### Inflammation in Brains from Alzheimer's Disease and Schizophrenia

Alzheimer's disease (AD) is a neurodegenerative disease with progressive dementia. β-Amyloid peptides that are formed in AD as insoluble aggregates ("senile plaques") are believed to cause neuronal and vascular damage. The amyloid deposits have been linked to oligodendrocyte damage in the white and gray matter[96]. These deposits can induce glial cells to produce cytokines[97]. Postmortem studies of the brain in individuals with AD demonstrated the presence of proinflammatory cytokines in senile plaques and neurofibrillary tangles. *In vitro*, the processing of β-amyloid precursor protein is affected by proinflammatory IL-1β. Fibrillar β-amyloid, in turn, promotes the production of proinflammatory cytokines by microglial and monocytic cell lines. Several studies using transgenic mice with systemic overexpression of IL-6 revealed the development of astrogliosis and chronic neurodegenerative disorders as well as age-related learning deficits in correlation to high IL-6 levels in the brain. In a rodent model, the blockage of TNF-α activity reduced cognition deficits evoked by intracerebroventricular injection of β-amyloid[98]. In a recently published cohort study including more than 2,800 dementia-free participants under continuous surveillance for the onset of neurologic disorders, those who had higher risk to develop AD also had high levels of IL-1β and TNF-α produced spontaneously by their cultured PBMCs[99]. It is possible that circulating cytokines and PBMC production of cytokines could predict the onset of AD in individuals free of clinical symptoms of the condition.

There is mounting evidence of increased expression of chemokines and chemokine receptors in AD. Altered function of CCR2 accelerates early disease progression and impairs microglial recruitment in a transgenic mouse model of AD, and AD mice deficient in CCR2 accumulated β-amyloid earlier and died prematurely[100]. Increased CXCR4 has been described in the AD brain[101], although no change in levels of expression compared to normal brain has been reported for other receptors, i.e., CXCR2[102]. In a study on a mouse model of AD, down-regulated CXCL12/CXCR4 was found to be associated with impaired learning[103]. An impact of chemokines in AD may be stage dependent as CCL2 was found in mature, but not immature, senile plaques[104]. Although several chemokines or chemokine receptors have been found up-regulated in the AD brain, whether they exacerbate AD or are protective is not yet conclusive from the present data[105].

Schizophrenia is a disabling mental disorder with symptoms such as auditory hallucinations, disordered thinking, apathy, etc. Evidence of a role for white matter involvement is rapidly accumulating. In a study using diffuse-weighted imaging of the brain in schizophrenic patients and healthy controls, cortical white matter microstructure disruption is described in different areas of the brain[106]. Significantly reduced staining of MBP has been found in a study on postmortem brain sections from individuals with schizophrenia compared to controls[107]. Evidence is also mounting that alterations in astrocyte functionality play a crucial role in the pathogenesis of schizophrenia. Traditionally, schizophrenia is regarded as a transmitter-based disorder, but recent evidence from clinical data supports a potential pathogenic role of elevated cytokine expression. Both childhood and adult schizophrenia have been shown to be accompanied by elevated expression of IL-1, IL-6, and TNF-α in the CSF[108,109]. With the exception of one study of a Japanese population regarding the role of cytokines[110], a majority of studies has consistently found altered cytokine or cytokine receptor expression in patients with schizophrenia[111,112], along with decreased expression of oligodendrocyte/myelin-related genes in postmortem brains[113,114]. An inflammatory process involved in schizophrenia is also indicated by data on the CCR3 ligand CCL11 being increased in hospitalized schizophrenic patients compared to control patients[115]. A case control study on a genetic variant of monocyte chemoattractant protein (MAP) 1 has not revealed linkage to the pathogenesis of schizophrenia, but to drug-resistant symptoms in schizophrenic patients[116]. A study on a 32-bp deletion in CCR5 suggested an association with susceptibility for late-onset schizophrenia[117], although it is not clear whether the CCR5 32-bp deletion allele may delay the onset of schizophrenia without affecting disease susceptibility.

### Traumatic CNS Injury Causes Inflammation and WMD

Traumatic injury of the CNS damages both neurons[118] and white matter, producing responses with acute and protracted time courses[119,120]. Modern MRI studies revealed that both gray and white matter are affected by trauma, with some studies finding a disproportionately greater loss of white matter damage[121]. Based on data in animal and human subjects, cellular apoptosis, necrosis, and inflammation have been suggested to cause acute and long-term damage of white matter tissue[122].

Altered cytokine levels identified in patients suffering from chronic spinal chord injury (SCI) after trauma support the assumption that chronic immunological activation occurs in these individuals. Although inflammation is a ubiquitous response after trauma in the CNS, the magnitude of cytokine increase is much higher in the spinal cord than in the brain, which could be attributed to the difference in the number and composition of invading leukocytes. It has been concluded from several clinical studies that the inflammation observed in brain trauma patients is a major contributing factor in the extent of brain damage and might serve as a target for therapeutic approaches.

In the CSF of adult and pediatric patients after traumatic brain injury (TBI), elevated concentrations of proinflammatory as well as anti-inflammatory cytokines have been found, i.e., IL-1β, IL-6, IL-8, TNF-α, IL-4, and IL-10[123,124,125,126,127,128]. The issue of whether specific cytokines may be used as prognostic criteria or as therapeutic targets remains controversial. In a recent study using brain microdialysis in patients after TBI, elevated levels of IL-1α, IL-1β, and endogenous IL-1 receptor antagonist, IL-1ra (receptor antagonist), were found and there was a significant correlation between increased IL-1ra and decreased intracranial pressure[129]. High concentrations of IL-1ra and high IL-1ra/IL-1β ratio were associated with improved outcome. Variants of the IL-1 gene family have been shown to have effects on clinical outcome after TBI in relation to clinical symptom scores of the Glasgow-Coma-Scale[130] and the onset of post-trauma intracranial hemorrhage[131], supporting the idea of a genetic influence on cytokine expression and clinical outcome.

Animal studies on the role of chemokines in the context of traumatic CNS injury have mainly been focused on CCL2. Only a few hours after stab implant into an adult mouse brain, levels of CCL2 increased markedly. *In situ* hybridization combined with immunohistology revealed that astrocytes are the main source of CCL2 under these conditions. Twenty-four hours after cryolesion, CCL2 gene expression was increased up to 20-fold on the ipsilateral side compared to controls. On the contralateral side, there was an increase of CCL2 RNA around 40% of that on the lesion side. In the spinal cord, CCL2 RNA up-regulation was also demonstrated about 50-fold compared to controls, coinciding with microglial reaction and accumulation of macrophages. In animal experiments, increased expression of the β-chemokine RANTES after rat brain stab wound injuries was found, peaking at day 1 and returning to baseline by day 7[132]. After cryoinjury, mice were described to have up-regulated RANTES starting within 1 h after injury and again returning to baseline within 1 week[133]. In a study in humans, RANTES was significantly higher in the serum of patients with severe brain injury than in non-TBI patients, and within the TBI patient group, it tended to correlate to severity of disease[134].

## LABORATORY MODELS OF INFLAMMATORY DEMYELINATION

### Experimental Allergic Encephalomyelitis (EAE)

EAE is the prototypical autoimmune model where paralytic disease is caused by an immune response to CNS antigens MBP, PLP, MOG, and S100 protein. It is hypothesized that in MS, exposure to environmental pathogens activates autoreactive T cells that recognize CNS autoantigens, leading to inflammation and demyelination. Utility of the EAE model for MS is promoted by clinical and histological similarities between MS and the various models of EAE. Adoptive transfer of MBP-specific T cells in the rat can induce an autoimmune-mediated disease of the CNS, but the disease is monophasic with little demyelination, unless modulated by cyclosporine[135]. The mouse CNS, however, is more sensitive to T-cell mediated damage[136]. Strain-specific mouse models commonly used include PLP-induced relapsing EAE and chronic progressive models of MOG-induced disease[137].

Although no single EAE model mimics the chronic disease courses of MS, and some differences exist between various EAE models, disease severity in EAE appears to be correlated with the recruitment of activated macrophages[138], which could also mediate axonal injury in the absence of extensive myelin loss[139]. However, despite the fact that EAE is unlike MS, the success of MS therapy based on EAE models has been promising, particularly in drugs designed for glucocorticoid[140] and antigen-specific therapies, e.g., glatiramer acetate[141,142] for clinical use.

EAE models are also being used to study chemokine function and to assess efficacy of antichemokine therapies. Histochemical analyses performed in EAE show that CCL2, CCL5, CCL19, CCL20, CXCL1, and CXCL10 are expressed in reactive astrocytes, while CCL1, CCL5, CCL19, and CCL22 are expressed in macrophages[143,144,145,146,147,148,149,150]. Treatment of an EAE model with AMD3100, an inhibitor of CXCR4, promoted the migration of infiltrating leukocytes into the CNS, leading to exacerbation of disease, indicating an anti-inflammatory role for CXCL12, the ligand for CXCR4[76]. The use of knockout animals in EAE has revealed that CCR8[149], CCR1[151], and CCR2[152,153,154] contribute to disease severity, while CXCR3-deficient mice show increased severity of EAE. Despite very promising results with chemokine knockout animals, including resistance to EAE, retardation in its progression, and reduced severity of disease, the high degree of redundancy of chemokine ligands and receptors has led to compensation. As a result, it has become difficult to suppress disease with one agent against a specific chemokine or receptor[155], and combination therapy may be required.

In anticytokine therapy, TNF blockade was effective in EAE[156], but human treatment with monoclonal antibodies or recombinant TNF receptor was unsuccessful, and even resulted in exacerbated inflammation[157]. Nonetheless, EAE models have helped immensely in the understanding of the basis of autoimmune disease. Because T(h)1 and T(h)17 T lymphocytes are implicated in EAE, the cytokines involved have been identified to be IFN-γ and IL-17, although the latter appears to be more important[158]. Thus, EAE models reproduce some aspects of brain inflammation in MS and remain useful in the testing of anti-inflammatory therapies and to test novel therapeutic targets, such as proteins of the blood coagulation cascade, which were identified in a recent study via proteomic screening of histologically characterized MS brain lesions[159].

### Cuprizone Demyelination

The oral administration of the copper chelator cuprizone (bis-cyclohexanone-oxaldehydrazone) produces copper deficiency and demyelination, and has been used extensively to induce fairly specific ablation of white matter tissue[160]. This demyelination appears to occur without damage to other cell types in the CNS other than oligodendrocytes, with lesion sites large enough to allow detection of changes in cellular, molecular, and biochemical characteristics. As with the EAE model, the extent of damage is dependent on species. Rats and guinea pigs show spongiform encephalopathy, whereas mice exhibit severe demyelination in a time frame of several weeks[161]. When 8-week-old C57Bl6 mice are fed with 0.2%

cuprizone, mature oligodendroglia are specifically ablated, and believed to be closely followed by recruitment and activation of microglia and peripheral macrophages, leading to phagocytosis of myelin. This inflammation is reported to occur in the absence of T lymphocytes and in the presence of an intact BBB. The toxicity of cuprizone is reversible, such that removal of the toxin from rodent diet is used to determine changes during remyelination, although both demyelination and remyelination occur simultaneously. While it had been presumed that copper deficiency was primarily responsible for the lesions, it was also noted that copper administration failed to reduce cuprizone toxicity[160], and the precise mechanism of demyelination/remyelination remained unexplained. A recent study utilizing IFN-γR–/– mice subjected to cuprizone demyelination showed delayed demyelination, indicating that IFN-γ was involved in tissue damage. Indeed, IFN-γ RNA was only detected in the corpus callosum at 2 weeks of cuprizone feeding, and not in control mice, indicating that IFN-γ contributes to demyelination[161]. In addition, IFN-γR–/– mice showed greater numbers of NG2+ OPCs and delayed microglial/macrophage infiltration in response to cuprizone feeding, although astrogliosis in the lesions was not affected by the absence of IFN-γR. Evidence for inflammation in this model of toxic demyelination has been steadily mounting. Cuprizone-induced lesions in IFN-β null mice were found to remyelinate more rapidly, concomitant with an increased number of OPCs within the demyelinated lesion[162], suggesting that elevated IFN-β prevents OPC recruitment and proliferation during remyelination. More compelling support for the role of proinflammatory cytokines was presented by the group of Pasquini et al.[163]. These studies, along with those of Cammer[164], indicated that the demyelinating effect of cuprizone is initiated indirectly through secretion of inflammatory cytokines such as IFN-γ and TNF-α. Application of cuprizone or sublethal doses of TNF-α and IFN-γ alone to cultured O4+ oligodendroglia did not decrease cell viability. Combination of cuprizone with cytokines dramatically lowered cell viability, which was preceded by the observation of mitochondrial abnormalities. Treatment with minocycline, which inhibits microglial activation, transmigration of T lymphocytes, and synthesis of MMPs, effectively diminished the number of CD11b+ activated microglia *in vivo*, and surprisingly prevented demyelination under cuprizone administration[163]. This body of recent evidence points toward the widespread involvement of cytokines and cellular interactions (see below) in the generation and repair of white matter lesions.

### Hypoxia/Ischemia

There is increasing evidence that postischemic inflammation plays an important role in brain ischemia. Rat models for hypoxia/ischemia have been established using carotid ligation and exposure to hypoxia (6–8% oxygen) for 2–24 h. Both acute and prolonged inflammatory responses can be observed, which are characterized by production of inflammatory cytokines in the brain and by activation of resident glial cells. In rodents, TNF-α mRNA increases within 1 h after brain ischemia in the ischemic injury zone followed by expression of TNF-α protein within 2–6 h[165]. Up-regulation of TNF-α and of TNF-α-R1 was detectable within 4–6 h of ischemia in astrocytes and microglia[166]. TNF-α can aggravate postischemic brain damage by its proinflammatory and procoagulant effects on endothelium, leading to impaired microcirculatory brain perfusion[167]. In contrast, an involvement of TNF-α in the signaling that regulates tolerance to brain hypoxia and ischemia has been shown in stroke animal models and in primary cultures of astrocytes[168]. Also in a neonatal rat model, hypoxia/ischemia of the brain led to transient stimulation of IL-1β mRNA and TNF-α mRNA expression[169]. Moreover, astrogliosis was found in this perinatal stroke model by increases in GFAP mRNA expression and GFAP immunoreactivity detected in the first 2 weeks after hypoxic/ischemic injury. The expression of mRNA and protein for IL-18 in homogenates of neonatal rat brains increased over weeks after hypoxia/ischemia[170]. IL-18 protein was highly expressed in microglia after hypoxia/ischemia *in vivo*. In IL-18 deficient mice, infarct volume was reduced by a fifth compared with wild-type mice. In a different study on inflammatory effects of hypoxia/ischemia in neonatal rats, treatment with hypothermia reduced IL-18 expression and suppressed microglial activation[171]. Interestingly, pre-exposure to mild hypoxia (e.g., 8% oxygen for 3 h) protects the brain of neonatal and adult rats against combined hypoxia/ischemia later on[172]. An anti-inflammatory mechanism seems likely to be involved in protection by this “preconditioning” because it reduced proinflammatory markers such as nuclear factor kappa B (NFκB) and microglial activation[173]. Neuroprotection by anti-inflammatory treatment has also been observed using neutralizing IL-1 antibody[174] and IL-10[31]. Administration of exogenous erythropoietin (EPO) starting 24 h after hypoxia/ischemia also has been shown to be neuroprotective in neonatal rats by preventing the rise in IL-1β and attenuating infiltration of leukocytes into the hypoxic/ischemic brain region[175].

### Intrauterine/Perinatal Infection

In animal models of neonatal infection, the endotoxin lipopolysaccharide (LPS) derived from various *E. coli* strains is often used to study inflammation in the context of bacterial infection. High systemic doses of LPS can induce symptoms of septic shock, i.e., hypotension, hypoglycemia, lactic acidosis[176,177], and can lead to white matter lesions. In the brains of 5-day-old rats, intracranial application of LPS leads to marked increase in TNF-α and IL-1β after a few hours and to white matter rarefaction after several days. Coadministration of IL-1ra with LPS significantly attenuated LPS-induced white matter injury, supporting the potency of IL-1β to cause WMD[178]. In a mouse model using LPS administration *in utero* to mimic chorioamnionitis, TNF-α and IFN-γ were significantly increased in the fetal brain after the stimulus compared to controls[179]. The impact of prenatal inflammation on white matter development in newborns is signified by experiments showing that newborn rats have increased intracranial IL-1β and TNF-α, and weaker MBP expression if their mothers prenatally received systemic LPS injections[180].

An intracranial increase of cytokines after LPS exposure is mainly assigned to activated CNS microglia and to systemic inflammation with ruptured BBB. Microglia express Toll-like receptor 4 (TLR-4), which has recently been identified as a receptor for LPS. In response to LPS receptor binding, TLR-4 and myeloid differentiation-2 (MD-2) form a receptor complex preceding different intracellular events[181]: an early response dependent on myeloid differentiation primary response gene 88 (MyD88) and MyD88-like adapter (Mal) leading to the activation of NFκB, and a later response to LPS using the Toll/interleukin-1 receptor (TIR)–domain-containing adapter-inducing IFN-β (TRIF) and TRIF-related adapter molecule (TRAM), which again leads to the late activation of NFκB and to interferon-responsive factor 3 (IRF3). Mainly, the later response causes induction of cytokines, chemokines, and transcription factors. TLR-4 seems to be crucial for LPS-induced neuronal degeneration[182]. Activated microglia release so-called “Golli proteins”, which enhance proliferation of OPCs at low concentrations of LPS, but decrease proliferation at higher concentrations of LPS[183].

The role of activated microglia in LPS-associated brain inflammation and WMD has been used for neuroprotective strategies using minocyclin as an inhibitor of microglia. In one study, the coadministration of minocyclin inhibited activation of microglia as assessed by cell shape and lectin histochemistry, and attenuated LPS-associated loss of late oligodendroglia precursor cells[184]. For neuroprotective treatment, EPO has been demonstrated to decrease WMD in newborn rats after perinatal maternal exposure to LPS[180]. The exact mechanisms by which EPO prevents WMD is not clear, but inhibition anti-inflammatory effects can be assumed given the fact that proinflammatory cytokines are decreased after administration of EPO in this model.

### Hyperoxia

Using a novel approach exposing Wistar rat pups to high oxygen concentrations, it was demonstrated that hyperoxia of 80% oxygen at postnatal day 6 for 24 h causes damage in the developing white matter, which was associated with increased apoptosis, oxidative stress, and acute inflammation[185]. Brain damage can be detected by increased silver staining in the cortical layers (Fig. 2A). Elevated expression of proinflammatory cytokines in the brain included IL-1β, IL-18[186], and IFN-γ. Systemic administration

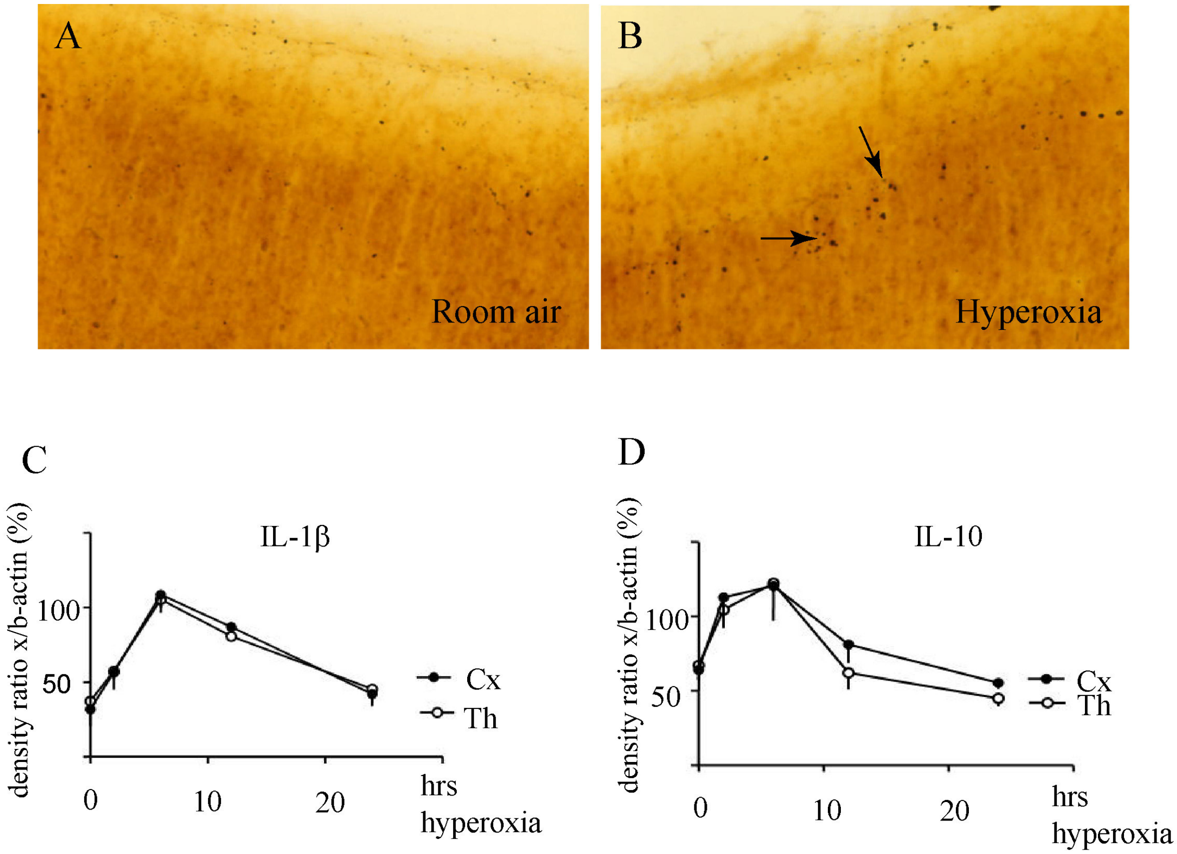

**FIGURE 2.** Effects of hyperoxia on cellular integrity and cytokine expression in the neonatal brain. (A,B) Cell damage indicated by silver staining (arrows) in cortex of Wistar rat pups at postnatal day 7 (P7) is increased after 24 h hyperoxia (B) compared to controls at room air (A). (C,D) Reverse transcription-quantitative polymerase chain reaction (RT-qPCR) analysis of proinflammatory IL-1β (C) and anti-inflammatory IL-10 (D) RNA levels in two regions of the P6 brain (Th = thalamus; Cx = cortex). Both cytokines are increased during exposure to hyperoxia, showing a peak at 6 h. Gene-specific qPCR values were normalized and expressed as percent of actin values. Cytokine levels in room air controls were undetectable.

of IL-18 binding protein to antagonize IL-18 action led to a significant decrease in brain damage measured by silver staining. Not only is proinflammatory cytokine expression altered, but anti-inflammatory IL-10 mRNA expression is also markedly increased after 6 h of exposure to hyperoxia (Fig. 2B). Moreover, mice deficient in IL-1β receptor-associated kinase-4 (IRAK-4) were resistant to oxygen-mediated neurotoxicity, indicating an important role for these proinflammatory cytokines in hyperoxia-induced brain damage. *In vitro* experiments showed that preoligodendrocytes produced significantly less MBP after exposure to hyperoxia[187]. In a model using hyperoxia of 100% oxygen in newborn sheep, brain tissue levels of mRNAs for IL-1β, TNF-α, IL-18, IL-6, and IFN-γ are elevated compared to sheep maintained in room air[188]. It can be inferred that high oxygen concentrations contribute to cerebral inflammation, hypomyelination, and WMD in preterm infants, thereby predisposing the neonate to long-term disability.

### Traumatic CNS Injury

In experiments on adult rats, TBI induced the release of IL-1α and IL-1β from neurons and astrocytes. IL-1 appears to regulate molecules involved in the development of injury. Intraventricular administration of antibodies to IL-1α and IL-1β before brain trauma significantly attenuated brain damage[189], possibly by inhibiting the expression of MMP-9 and intracellular adhesion molecule-1 (ICAM-1), which regulate leukocyte trafficking across the endothelium. In an adult mouse model of closed head injury, significantly elevated IL-18 protein levels in injured brains were detected at 7 days after trauma[190]. Head trauma to newborn rodents is linked to increased intracerebral synthesis of IL-1β and IL-18, and inhibition of IL-18 by IL-18-binding-protein attenuated apoptotic cell death. In IL-1R–deficient mice, astrogliosis measured by increased GFAP production due to brain trauma was inhibited compared to wild-type mice, emphasizing a role of IL-1R in traumatic astroglia activation[191]. Experiments in adult rats showed that inflammatory responses after TBI occur both locally in the brain and systemically as determined by serum levels of cytokines: proinflammatory IL-1β and anti-inflammatory IL-10 increase markedly within a few hours only in the brain, while TNF-α is elevated only in the serum, but not in the brain[192]. Elevated mRNA levels of TNF-α and IL-6 were found in adult rat brains 6 h after TBI[193]. In TNF-α knockout mice, decreased brain lesions and improved motor performance was observed several days after TBI[194].

It was demonstrated in adult rats that systemic administration of high-dose recombinant erythropoietin (rhEPO) led to an attenuation of post-TBI IL-1β and TNF-α expression, and reduced cell apoptosis compared to controls without rhEPO[195]. In addition, the intraperitoneal administration of progesterone attenuated the post-traumatic increase in the expression of inflammation-related factors NFκB and TNF-α, but not reactive GFAP. These changes, however, were associated with reduced lesion volume, suggesting improved outcome[196]. In these studies, modulation of inflammatory response after injury mitigates the severity of CNS damage.

### SIGNALING PATHWAYS OF CYTOKINE- AND CHEMOKINE-MEDIATED RESPONSES

Cytokines exert a diverse array of effects and even dissimilar cytokines can elicit similar cellular responses. Functional redundancy results, in part, from similarities in molecular structure within receptor subunits and downstream effector mechanisms. Oligodendrocyte lineage cells express multiple chemokine receptors and myriad cytokine receptors, including those of the Th1 and Th2 categories[197], and space constraints necessitate many omissions in order to focus this discussion on the Th1-mediated cascades.

#### IL-1 Signaling

The proinflammatory cytokine IL-1 is one of the most studied cytokines to date. In oligodendrocyte lineage cells, IL-1β elicits mixed responses. Acutely, IL-1β induced ceramide accumulation, resulting in acute apoptotic death, but chronic treatment was not lethal[198]. Instead, IL-1β was found to promote oligodendrocyte differentiation, inhibiting expression of the progenitor marker PDGFRα[8]. The effects of IL-1 are mediated by the IL-1 type receptor, which contains immunoglobulin-like domains and functions in concert with an accessory protein, IL-1 receptor accessory protein (IL-1RacP), to form a complex. The pathway involves the activation of IRAK-1 and IRAK-2, leading to the downstream induction of NFκB and activator protein 1 (AP-1) transcription factor complexes via activation of TNF receptor associated factor-6 (TRAF6) and TGF-β–activated kinase (TAK-1) and TAK binding protein, TAB-1[199] (Fig. 3A). The proteins TAB and TAK link TRAF6 to IκB kinase (IKK) in IL-1 signaling[200]. Inactive NFκB proteins are heterodimers or homodimers sequestered in the cytoplasm by binding IκB proteins. A multitude of extracellular signals can lead to the phosphorylation of IκB proteins by IKK. TAK1 can directly phosphorylate IKK and mitogen activated protein kinase kinase 6 (MKK6), leading to the activation of both Jun N-terminal kinase (JNK) and NFκB signaling pathways. Phosphorylated IκB is then degraded by the proteasome, unmasking a nuclear localization signal of the associated NFκB, resulting in its translocation to the nucleus and binding to promoters of target genes. In oligodendrocytes, IL-1β demonstrated selective and IL-1R–dependent effects on the mitogen-activated protein kinase (MAPK) pathway in OPCs, activating p38MAPK while suppressing p44/42 phosphorylation[8].

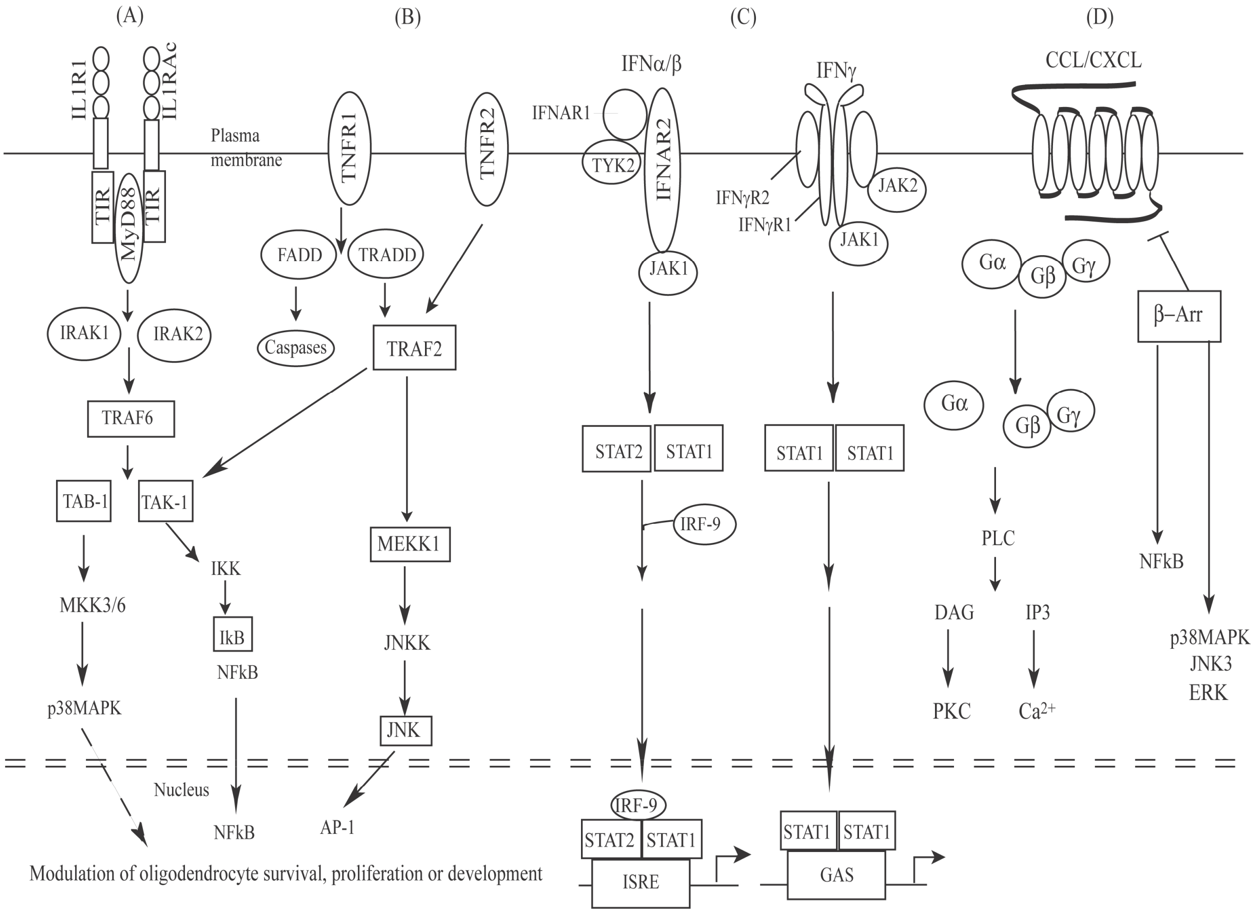

**FIGURE 3.** Schematic representation of signaling pathways utilized by chemokines and selected cytokine receptors. The cytokine receptors are related to the mechanisms by which each cytokine transmits intracellular signals. (A–C) Receptors for Th1 proinflammatory cytokines; the IFNs, TNF, and IL-1 are shown. Cytokine signaling events begin with receptor-ligand interaction at the plasma membrane, leading to the activation of kinases (JAK, IRAK, MEKK) and subsequent phosphorylation of substrates that bind specific downstream effectors, which eventually affect nuclear transcription factors (e.g., NFκB) to control gene expression. (D) Chemokines signal through G-protein-coupled seven transmembrane receptors. Dissociation of G proteins (Gα, Gβγ) signals to downstream effectors phospholipase C (PLC), diacylglycerol (DAG), protein kinase C (PKC), inositol 1,4,5-trisphosphate (IP3), beta-arrestin (β-Arr), extracellular signal-regulated kinase (ERK).

### TNF Signaling

TNF receptors are capable of many cellular responses, but are chiefly associated with the induction of cell death mechanisms[201]. TNF-α binds to two distinct, but structurally related, receptors, a 55-kDa TNFR1 and a 75-kDa TNFR2[202,203], which are differentially regulated on various cell types in normal and disease states[204], and are activated with different kinetics[205]. TNFR1 is the receptor isoform primarily responsible for hyper-responsiveness, and, while the consequences of TNFR2 signaling are less well characterized, TNFR2 has been shown to mediate signals that promote tissue repair and angiogenesis[206]. Activation of TNFR1, or TNFRp55, results in recruitment of TNFR1-associated death domain (TRADD) protein[207]. TRADD can also interact with TRAF proteins or with Fas-associated protein with death domain (FADD), which signal the apoptotic protease cascade[208,209]. The family of TRAF adaptor proteins interacts with both TNFR1 and TNFR2. TRAF molecules all contain RING (Really Interesting New Gene) finger and zinc finger motifs in their N-terminus, with a TRAF domain at the C-terminus. The conserved TRAF region allows adaptors to interact with cell surface receptors or other signaling molecules. TRAFs serve a wide variety of receptors involved in regulating stress responses, cell survival, and cell death. To date, at least six mammalian TRAFs have been identified. It has been shown that the proliferative effects of TNF-α on OPCs are mediated by TNFR2[9], which signals primarily through TRAF2 proteins. TRAF2 also interacts with many downstream molecules which belong to the family of MAPK kinases (MEKK). Indeed, NFκB, which is activated by TRAF2, is important for the cytoprotective effects of TNF[210]. It is well established that TRAF2 can regulate AP-1 transcriptional activators[211]. There is evidence that TNF-α activates c-Jun, a component of the AP-1 complex, via JNK phosphorylation (Fig. 3B)[212]. Since AP-1 transcription stimulates cell cycle genes such as cyclin D, which regulates G1/S checkpoint control[213,214], AP-1 activation by TNF-α is in agreement with the proliferative response of OPCs to TNF-α[16].

### IFN Signaling

The type I (α, β, ω) and type II (γ) IFN receptor families modulate gene expression via a simple direct signaling pathway containing Janus tyrosine kinases (JAK), and signal transducers and activators of transcription (STAT) (Fig. 3C). Additional pathways activated by IFN-γ that pertain to endoplasmic reticulum stress and apoptosis in oligodendrocytes is beyond the scope of this article. The JAK/STAT proteins also mediate signaling events of many other cytokine receptors, including IL-6, IL-10, LIF, and CNTF. IFN receptors have an extracellular ligand-binding domain and intracellular kinase domain that are activated by ligand-induced dimerization. Type I IFN receptors comprise two subunits, AR1 and AR2, which both possess ligand-binding properties. IFN-α binds two JAK kinases, tyrosine kinase 2 (TYK2) and JAK1. This binding of type I IFNs activates JAK, STAT1, and STAT2, which stimulates the transcription of genes containing the IFN-stimulated gene response element (ISRE). JAKs are coupled with the intracellular domain of cytokine membrane receptors. There are seven members of the mammalian STAT family, all featuring an SH2 domain that mediates interaction with JAKs and other STAT proteins. IFN-γ receptors consist of two IFN-γR1 chains, which prompt the association of two IFN-γR2 subunits. R1 and R2 are constitutively associated with inactive forms of the tyrosine kinases JAK1 and JAK2, respectively. Both JAKs transactivate each other to recruit STAT1 to IFNR1. Activated STAT1 dimers translocate to the nucleus to initiate transcriptional responses through the binding of cognate response elements, such as the gamma-activation sequence (GAS). In ischemia, a strong increase in the expression of STAT3 and STAT1 occurs in reactive astrocytes and microglia, and even in neurons, although its function is less understood. Additional activities of STAT proteins include c-Jun recruitment and p300/CREB-binding protein (CBP) binding[215]. IFN-β inhibited the differentiation of OPCs only in the presence of astrocytes and microglia, without any change in proliferation or survival, arguing that the effect of IFN-β was likely to be indirectly mediated by other glial cells[216]. However, IFN-γ stimulates binding of STAT1 to the GAS site (Fig. 3C) on the TNFR1 promoter directly in cultured oligodendrocytes[217]. Up-regulation of the expression of TNFR1 RNA in this manner likely underlies the synergism between TNF-α and IFN-γ to inhibit the proliferation of OPCs and their differentiation into mature oligodendrocytes[25]. The effect of IFN-γ has been shown to be specific for mature oligodendrocytes, and notably spares immature progenitors, without producing apoptosis. There is now accumulating evidence that multiple signaling pathways are activated by IFNs and that IFN-γ–inducible transcription of certain genes is STAT independent[218]. The IKK complex has been implicated in IFN signaling, although its function appears independent of NFκB, suggesting additional functions for these kinases.

### Chemokine Signaling

Chemokine receptors are G-protein-coupled receptors (GPCRs) with seven transmembrane domains that are highly conserved. GPCRs represent the most widely targeted pharmacological protein class. Upon binding cognate receptors, chemokine response is mediated by G proteins and can be blocked by pertussis toxin, indicating mediation by the $G\alpha_{i/o}$ class of G proteins. The G-protein complex exchanges guanosine diphosphate (GDP) for guanosine triphosphate (GTP) and dissociates upon activation of the receptor into Gα and Gβγ. Gα inhibits adenylyl cyclase and cyclic AMP formation and protein kinase A phosphorylation, and can activate Src tyrosine kinase[219]. The release of free Gβγ subunits of G protein is required for chemokine-induced chemotaxis[220]. Gβγ activates the small G protein Ras, which in turn directly activates PI3K, promoting the conversion of PIP2 into PIP3[221,222]. IP3 binds its specific receptor in the endoplasmic reticulum and releases calcium from intracellular stores[223]. Gβγ also activates PLC isoforms, which in turn stimulate diacylglycerol production and PKC activation. Agonist-bound GPCRs are subjected to desensitization or down-regulation, which decreases the ability to reinitiate signaling. The phosphorylation of GPCRs by serine/threonine GRKs (GPCR kinases) increases the affinity of the GPCR for arrestins, which prevents the GPCR from associating with its G protein[224]. Receptor-bound β-Arr act as signaling scaffold for MAPK cascades, leading to robust activation of JNK3, ERK, and p38 (Fig. 3D)[225,226,227,228]. OPCs, when stimulated by CXCL12, show intracellular calcium elevation via the activity of CXCR4[229]. Stimulation of OPCs with CCL11, which activates CCR3, also showed an increase in release of intracellular calcium[230]. Many cross-regulatory mechanisms of related pathways add to the complexity of chemokine-mediated signal transduction, e.g., CXCL12 activates NFκB and induces cell death in primary astrocytes[42]. The functional features of chemokine signaling in glial cells is currently not as well documented as in immune cells, and clearly, when more is learned in the CNS, such as the nature of receptor oligomerization, which could alter the signaling properties of the receptors, the development of novel pharmacological modulators could then lead to more effective targeting of receptor function.

### CLOSING REMARKS

The discovery of new cytokines has been growing with rapidly accumulating genomic information, and, given the cellular heterogeneity of neural tissue, the increased complexity of cytokine action in the CNS will continue to evolve. There is now ample evidence that most cytokines, by virtue of their pleiotropic nature, and despite classification as pro- or anti-inflammatory, contribute to the pathophysiology of CNS trauma and immune activation with both beneficial and detrimental effects, causing cell damage and death on the one hand, and promoting tissue repair on the other. As a result, global interference with cytokine function or signaling has produced conflicting outcomes. The vulnerability of oligodendrocyte lineage cells to insults is widely believed to be stage dependent, and many approaches to mitigate WMD demand knowledge of the mechanisms of cellular dysfunction in both progenitors and mature myelinating cells. With the advent of broad-based gene expression profiling, concerted efforts in the identification of novel target markers, together with improved monitoring of immunomodulatory treatment and understanding of cytokine interactions, will help in the design of therapeutic strategies that can safely traverse the divide between bench and bedside.

### ACKNOWLEDGMENTS

The authors of this review are grateful to Dr. Vittorio Gallo for suggestions and support through National Institutes of Health grant R01NS1056427. TS is additionally supported by "Sanitätsrat Dr. Emil Alexander von Huebner und Gemahlin-Stiftung", Germany.
